# Autophagy Is Involved in the Cardioprotection Effect of Remote Limb Ischemic Postconditioning on Myocardial Ischemia/Reperfusion Injury in Normal Mice, but Not Diabetic Mice

**DOI:** 10.1371/journal.pone.0086838

**Published:** 2014-01-23

**Authors:** Zhihua Han, Jiatian Cao, Dongqiang Song, Lei Tian, Kan Chen, Yue Wang, Lin Gao, Zhaofang Yin, Yuqi Fan, Changqian Wang

**Affiliations:** Department of Cardiology, Ninth People’s Hospital, Shanghai Jiaotong University Medical School, PR China; UAE University, Faculty of Medicine & Health Sciences, United Arab Emirates

## Abstract

**Background:**

Recent animal study and clinical trial data suggested that remote limb ischemic postconditioning (RIPostC) can invoke potent cardioprotection. However, during ischemia reperfusion injury (IR), the effect and mechanism of RIPostC on myocardium in subjects with or without diabetes mellitus (DM) are poorly understood. Autophagy plays a crucial role in alleviating myocardial IR injury. The aim of this study was to determine the effect of RIPostC on mice myocardial IR injury model with or without DM, and investigate the role of autophagy in this process.

**Methodology and Results:**

Streptozocin (STZ) induced DM mice model and myocardial IR model were established. Using a noninvasive technique, RIPostC was induced in normal mice (ND) and DM mice by three cycles of ischemia (5 min) and reperfusion (5 min) in the left hindlimb. In ND group, RIPostC significantly reduced infarct size (32.6±3.0% in ND-RIPostC vs. 50.6±2.4% in ND-IR, *p*<0.05) and improved cardiac ejection fraction (49.70±3.46% in ND-RIPostC vs. 31.30±3.95% in ND-IR, *p*<0.05). However, in DM group, no RIPostC mediated cardioprotetion effect was observed. To analyze the role of autophagy, western blot and immunohistochemistry was performed. Our data showed that a decreased sequestosome 1 (SQSTM1/p62) level, an increased Beclin-1 level, and higher ratio of LC3-II/LC3-I were observed in ND RIPostC group, but not DM RIPostC group.

**Conclusions:**

The current study suggested that RIPostC exerts cardioprotection effect on IR in normal mice, but not DM mice, and this difference is via, at least in part, the up-regulation of autophagy.

## Introduction

Infarct size is a major determinant of mortality in acute myocardial infarction (AMI), a main cause of death worldwide, especially in the industrial countries. Limiting infarct size is the critical event to improve immediate and long-term outcome in patients with an acute coronary syndrome and to avoid heart failure [1]. Currently, the most effective way to limit infarct size is to reopen the “culprit” vessel and reperfuse the jeopardized myocardium with thrombolytic drugs or primary PCI as soon as possible [2]. Although reperfusion is undoubtedly beneficial, its detrimental effect on clinical outcome, including myocardial stunning, ventricular arrhythmias, and microvascular dysfunction, is still a major challenge for cardiologists.

Post ischemic conditioning is a protective strategy that attenuates myocardidal IR injury [3], [4], [5]. Zhao et al reported that post ischemic conditioning (three cycles of 30-s reperfusion and 30-s left anterior artery reocclusion) is as effective as pre-conditioning in reducing infarct size and preserving endothelial function in swine myocardial IR model [3]. Botker et al also indicated that, before primary percutaneous coronary intervention, remote ischaemic conditioning during evolving ST-elevation myocardial infarction increases myocardial salvage and has a favorable safety profile in a random trial [4].

Compared with local ischemic postconditioning, which is limited to coronary angioplasty patients, remote Limb ischemia postconditioning (RIPostC) is much easier to perform in the setting of AMI. Although this advantage makes RIPostC potentially applicable in clinical practice [5], more questions need to be answered before it can be widely used in clinical application.

First, what is the mechanism of RIPostC cardioprotective effect in IR injury? Very few reports have explored the mechanism involved in this cardioprotection activity. Okorie et al demonstrated that this protection is via inhibiting mitochondrial permeability transition pore, opening mitochondrial ATP-dependent potassium channels, and protecting endothelial function against IR injury [6]. However, other important mechanism may also contribute to this regulation process.

Macroautophagy (hereinafter referred to as autophagy) is an intracellular bulk process of self-digestion involving the lysosomal degradation of cytoplasmic organelles and macromolecules. It is important in regulating cell functions by maintaining cellular homeostasis, energy balance, and providing cytoprotective responses to adverse conditions [7]. It has been suggested that autophagy malfunction can induce the pathogenesis of diverse human diseases, such as liver disease, tumor, neurodegeneration disease, and aging [8], [9]. Moreover, recent clinical trials have demonstrated that induction of autophagy can rescue heart function in IR injury [10], [11], [12]. Considering the effect of RIPostC on IR injury, we hypothesized that autophagy is a major mechanism involved in RIPostC cardioprotection activity.

Secondly, is RIPostC only effective in some certain setting of subjects, such as DM patients? DM, a major risk factor for ischemic heart disease, is associated with increased adverse outcomes in terms of morbidity and mortality over the short and long term after a coronary artery event [13]. Although DM patients have been included in some clinical trials, no report of RIPostC cardioprotective effect on this setting has been illustrated.

In this study, we sought to determine: (1) Is autophagy pathway involved in the cardioprotection activity of RIPostC? (2) Does RIPostC offer potential benefit in ameliorating IR injury in murine myocardial IR model with or without DM?

## Materials and Methods

### Experimental Animals

All animals used in this study were received humane care in compliance with principles stated in the Guide for the Care and Use of Laboratory Animals, NIH Publication, 1996 edition. All protocols were approved by the Animal Care Committee of Shanghai Ninth Hospital, Shanghai Jiao Tong University School of Medicine. C57BL/6 mice (male, 8–12 weeks of age) were obtained from the SLAC Laboratory (shanghai laboratory animal center, Shanghai, China), and housed for two weeks as an acclimatization period before the experiment.

### Generation OF DM mice

Diabetes was induced in male C57BL/6 mice via a single dose intraperitoneal injection of 150 mg/kg Streptozocin (STZ, Sigma-Aldrich, USA, S0130) in 0.1 M citrate buffer (pH 4.1) one week prior to surgery [14]. Normal mice (ND, means non-diabetes) animals received an equal volume of citrate buffer. Development of the diabetes was confirmed by measuring blood glucose levels using a glucometer device (ACCU-CHEK® Performa, Roche) through sampling of blood with a small scratch in mouse tail. After 72 hours, the mice with blood glucose levels higher than 300 mg/dL were considered diabetic, and those with blood glucose levels lower than that were excluded from the experiment [15], [16].

### Induction of RIPostC

Using an open invasive technique, RIPostC was induced by 3 cycles of 5 min of left femoral artery occlusion by a microvascular clamp to occlude the femoral vessels under an operating microscope, followed by 5 min of reperfusion as described previously [16], [17]. Unilateral distal limb pallor was observed during occlusion, followed rapidly by brisk reactive hyperemia during reperfusion.

### Surgical Preparation and Induction of Ischemia Reperfusion

C57BL/6 mice on standard diet were subjected to myocardial IR at 8–12 weeks of age. Surgery was performed as previously described [18]. In brief, mice were kept in a 4% isoflurane anesthesia chamber with oxygen delivered through a nose cone and tracheal intubation. An 8-mm skin incision was made 2 mm from the left sternal border at the fourth intercostal space directed towards the left axilla. The left coronary artery (LCA) was identified after retraction of the left atrium and ligated 1 mm from the tip of the left atrial appendage with the use of a 7–0 suture on a tapered needle (add a reversible suture). Occlusion was confirmed by balancing of the LV myocardium below the suture. For animals undergoing a shame operation, a ligature was placed in a corresponding location but not tied. Mice were subjected to 30 minutes of transitory ligation followed by 3 h of reperfusion. Reperfusion was confirmed by visualization of the return of color reflecting blood flow in the previously pale region and by immediate electrocardiographic changes including resolution of ST segment elevation detected with the use of a base plate electrocardiographic system (VisualSonics).

### Study Groups and Experimental Protocol

**Figure 1:**
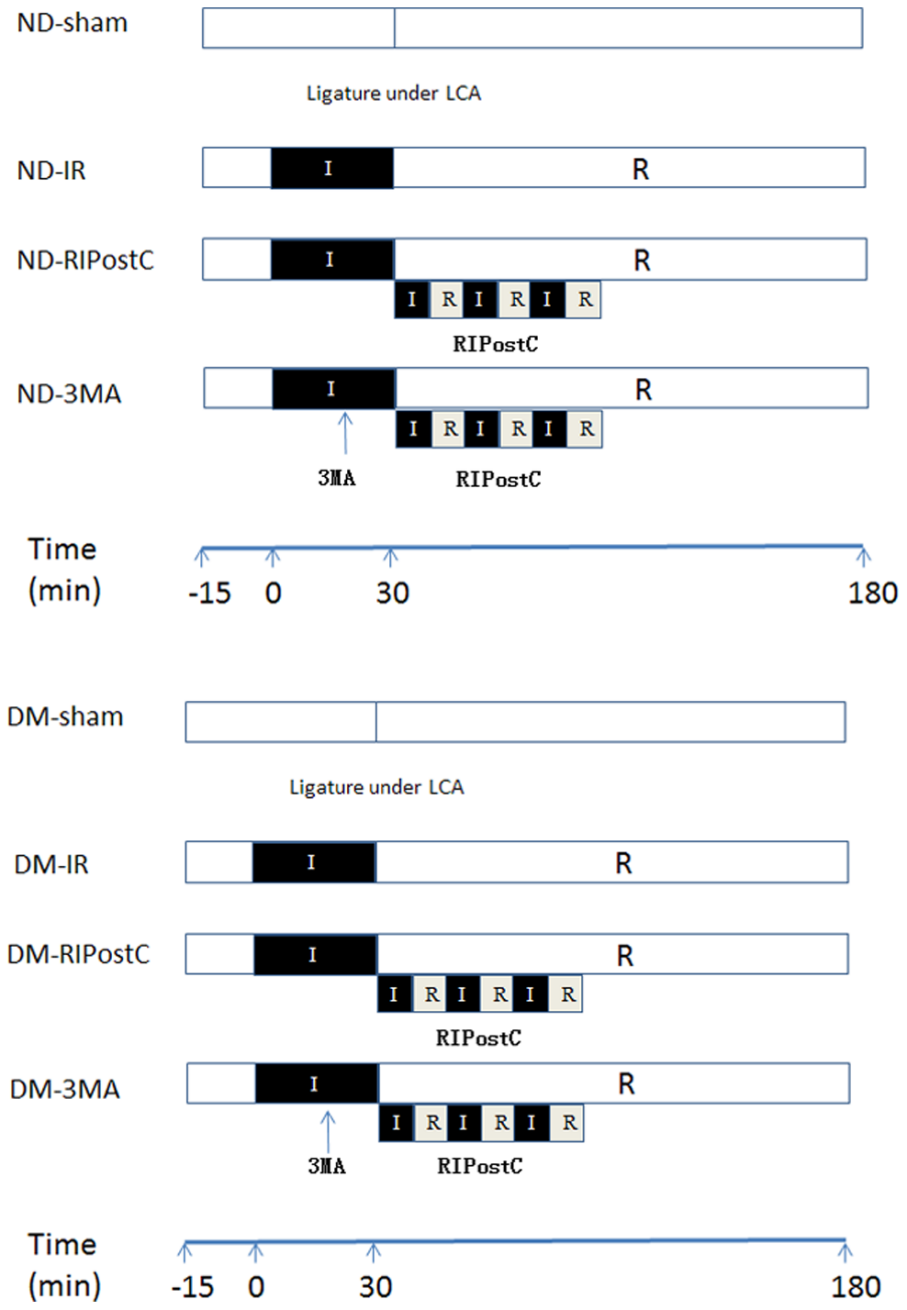
Experimental protocol. (1) ND-sh (n = 24), placing the ligature under LCA; (2) ND-IR (n = 30), occlusion of LCA for 30 min, was followed by 3 h of reperfusion; (3) ND-RIPostC (n = 30), three RIPostC cycles were applied at the onset of coronary reperfusion period; (4) ND-IR+RIPostC +3MA (n = 30), three RIPostC cycles were applied at the onset of coronary reperfusion period, and received 3MA treatment 10 min before coronary reperfusion; (5) DM-sh (n = 24), placing the ligature under LCA; (6) DM-IR (n = 30), occlusion of LCA for 30 min, was followed by 3 h of reperfusion; (7) DM-RIPostC (n = 30), three RIPostC cycles were applied at the onset of coronary reperfusion period; (8) ND-3MA (n = 30), three RIPostC cycles were applied at the onset of coronary reperfusion period, and received 3-MA treatment 10 min before coronary reperfusion.

Mice were randomly assigned to the following groups (Figure 1).

1. Nondiabetic shame group (ND-sh, n = 24), in which mice were placed the ligature under the LCA, and only underwent mobilization of the right femoral vascular bundle.
2. Nondiabetic IR group (ND-IR, n = 30), in which mice were subjected to 30-min coronary artery occlusion followed by 1, 2, or 3 h reperfusion.
3. Nondiabetic IR plus RIPostC group (ND-RIPostC, n = 30), in which mice were received left hindlimb intervention with 3 cycles of 5-min reperfusion followed by 5-min ischemia immediately at the onset of coronary reperfusion period.
4. Nondiabetic IR with RIPostC and 3-methyladenine (3-MA, an autophagy inhibitor) group (ND-3MA, n = 30), in which mice were received 3-MA 10 min before coronary reperfusion and left hindlimb intervention with 3 cycles of 5-min reperfusion followed by 5-min ischemia at the onset of coronary reperfusion.
5. Diabetic shame group (DM-sh, n = 24) in which mice were placed the ligature under the LCA, and only underwent mobilization of the right femoral vascular bundle.
6. Diabetic IR group (DM-IR. n = 30), in which mice were subjected to 30-min coronary artery occlusion followed by 1, 2, or 3 h reperfusion.
7. Diabetic IR with RIPC group (DM-RIPostC, n = 30), in which mice were received left hindlimb intervention with 3 cycles of 5-min reperfusion followed by 5-min ischemia at the onset of coronary reperfusion.
8. Diabetic IR with RIPostC and 3-MA group (DM-3MA, n = 30), in which mice were received 3-MA 10 min before coronary reperfusion and left hindlimb intervention with 3 cycles of 5-min reperfusion followed by 5-min ischemia at the onset of coronary reperfusion.

Autophagy inhibitor 3-MA (10 mg/kg, Sigma-Aldrich, M9281) was dissolved in sterile phosphate buffered saline (PBS) and administered via the intraperitoneal injection 10 min before coronary reperfusion [19].

### Measurement of Myocardial Infarct Size (IS) and Area at Risk (AAR)

Myocardial infarct size was assessed to measure the extent of IR injury. Infarct sizes (IS) were identified as described previously [18]. At the end of the experiment, the coronary artery was retied at the same site and 0.5 mL of 1% Evans blue was injected into the left cavity to identify the area at risk (AAR) as unstained from the blue, non-ischemic part of the myocardium. The hearts were then frozen at −20°C and thereafter cut into thin 5 slices (2 mm) from the apex to the base. The slices were incubated in 1% 2, 3, 5-triphenyltetrazolium chloride (TTC, Sigma-Aldrich) in phosphate-buffered solution, pH 7.4 for 10 minutes at 37°C. The heart slices were immersed in 10% formalin for 24 hours to identify viable myocardium as red stained, white necrotic (infarcted) tissue remains pale gray. The infarct area (characterized by absence of staining), noninfarcted AAR (characterized by bright red tissue staining), and the nonischemic ventricle (characterized by blue tissue staining) were photographed and measured. The extent of the area of necrosis was quantified by computerized planimetry (Image J 1.4) and corrected for the weight of the tissue slices. IS was expressed as the percentage of total weight of the LV AAR.

### Heart Collection

Mice in each group were euthanized 1, 2, or 3 hrs after the induction of myocardial reperfusion. After perfusion with PBS, the hearts were immediately harvested. The left ventricular (LV) was carefully separated from the right ventricle and atria. Then one cross-section of LV myocardial tissue at the level of the papillary muscles, approximately 5 mm, was collected and fixed in 4% formalin for the histology examination. The remaining LV tissue was frozen immediately in liquid nitrogen. Samples were stored at −80°C until use.

### Western Blot Analysis

For further biochemical analysis, Western blotting was performed on homogenates of LVs from C57BL/6 mice. Proteins prepared from mouse hearts were quantified by Bio-Rad protein assay. For immunodetection, 30 µg of crude lysates prepared as above were resolved on SDS-PAGE 10% denaturing gels (15% gels for LC3B) and transferred to nitrocellulose membranes. The membranes were blocked with 5% nonfat dry milk in TBST buffer (100 mM NaCl, 10 mM Tris-HCl, pH 7.4, and 0.1% Tween-20) for 1 h. The blots were then incubated with 1000-fold diluted primary antibodies against LC3B (Sigma-Aldrich, USA, L7543), Belcin1 (Proteintech, USA, 11306-AP), SQSTM1/p62 (Abcam, HK, Ab91526), phospho-AMPKα (Thr172) (Cell Signaling Technology, #2535), AMPKα (Cell Signaling Technology, #2532 ) and GAPDH (Proteintech, USA, 10494-1-AP) at 4°C overnight and then washed with TBST buffer at room temperature and incubated with appropriate peroxidase-conjugated secondly antibody (1∶5000 dilution). Immunoreactive bands were visualized by chemiluminescence (Odyssey Li-COR). Each immunoblotting experiment was repeated three times, and the results were averaged. To quantity the protein, band intensity was assessed by Quantity one 4.6.2 software.

### Echocardiography

The echocardiography analysis in animals was performed 3 h after reperfusion. Images were obtained using Acuson Sequoia 512 (Siemens company, Germany) equipped with a 15-MHz probe. The mice were lightly anaesthetized using 1.5% and restrained on a heated imaging table. The four limbs were attached to ECG electrodes and hairs on the chest were removed using Nair. Images were obtained from the B-mode parasternal long axis view, M-mode of the parasternal short-axis view. LV anterior and posterior wall dimensions during diastole and systole were recorded from three consecutive cycles in M-mode using methods adopted by the American Society of Echocardiography [18]. Fractional shortening was calculated from LV end-diastolic (EDD) and end-systolic (ESD) diameters using the equation of (EDD-ESD)/EDD. Heart rates were averaged over 10 cardiac cycles. All values were averaged over five consecutive cardiac cycles and measurements were analyzed by two independent researchers blinded to the treatment status.

### Immunohistochemistry Analysis

For Immunohistochemistry, heart tissues were fixed in 10% buffered formaldehyde solution and embedded in paraffin. In brief, serial sections were cut as 5 µm thickness using a microtome, dewaxed in xylene, and rehydrated in alcohol, and then endogenous peroxidase activity was blocked with 10% hydrogen peroxide in water for 5 min. The tissue sections underwent microwave antigen retrieval, then were blocked with 10% goat serum in PBS, and incubated with primary antibody (diluted 1∶50–1∶200) overnight at 4°C. Sections were incubated with secondary antibody for 1 h at room temperature, incubated with avidin-biotin complex for 1 h at room temperature, rinsed with PBS and then treated with 0.5 mg/ml 4, 6-diamidino-2-phenylindole (DAPI) to reveal immunoreactivity. The expression of LC3B, Beclin-1 and SQSTM1/p62 was evaluated in a semi-quantitative method. The primary antibodies used in this study were listed below.

LC3B: a mouse LC3B antibody, diluted 1∶50 (Sigma-Aldrich, L7543), cytoplasmic staining.

Beclin1: a pre-diluted mouse monoclonal antibody, diluted 1∶50 (Cell Signaling, #3495), cytoplasmic staining.

SQSTM1/p62, polyclonal antibody (Abcam, ab91526), cytoplasmic staining.

### Tissue Preparation for Transmission Electron Microscopy

After 3 h of reperfusion, small piece of myocardium sample from ND versus DM sh, IR, RIPostC, and 3-MA group were fixed in 4% glutaraldehyde overnight at 4°C. The tissue pieces were post-fixed in 1% osmium tetroxide for 60 min at 4°C before being dehydrated in a graded series of ethanol, and embedded in Spurr’s epoxy resin. Ultrathin sections (60–70 nm) were then cut with diamond knives and retrieved onto copper mesh grids. The sections were then contrasted with uranyl acetate and lead citrate. The Ultrathin sections were examined with CM-120 transmission electron microscope (PHILIP, The Netherlands) operating at 60 kV. Digital electron micrographs were recorded with a MegaView III CD using iTEM-SIS software (Olympus, Soft Imaging System GmbH, Germany). Autophagosomes were identified by transmission electron microscopy as previously reported [20], [21]. Image contrast was enhanced in Adobe Photoshop CS2.

### Statistical Analysis

All data are presented as mean ± standard error of mean (S.E.M.). One-way analysis of variance (ANOVA) was performed to test treatment effect. Difference between groups was determined using Tukey’s post-hoc test with *P*≤0.05 considering as significant.

## Results

### Characteristics of Animals

STZ-induced diabetic mice showed a higher mortality rate as 16 diabetic mice died during coronary reperfusion period. Totally 4 mice, in which blood glucose level was lower than 300 mg/dL after 1 week induction, were excluded from the further experiment.

Significant difference of body weight, heart weight, and preoperative serum glucose values were observed between ND group and DM group (Table 1).

**Table 1:** Baseline characters of ND and DM mice.

Groups	Blood GlucoseMg/dL	Body Weight(BW) g	Heart Weight(HW) g	HW/BW
ND(n = 23)	138.2±9.8	24.1±2.3	101.7±3.1	4.29±0.39
DM(n = 28)	416.3±23.5	19.3±1.4*	89.2±2.7*	4.64±0.33*

Abbreviation: SEM, standard error of the mean.

Data was expressed as mean±SEM.

* p<0.05 *vs* ND, ^#^P<0.01 *vs* ND.

### Effect of RIPostC on LV Systolic Function

**Figure 2:**
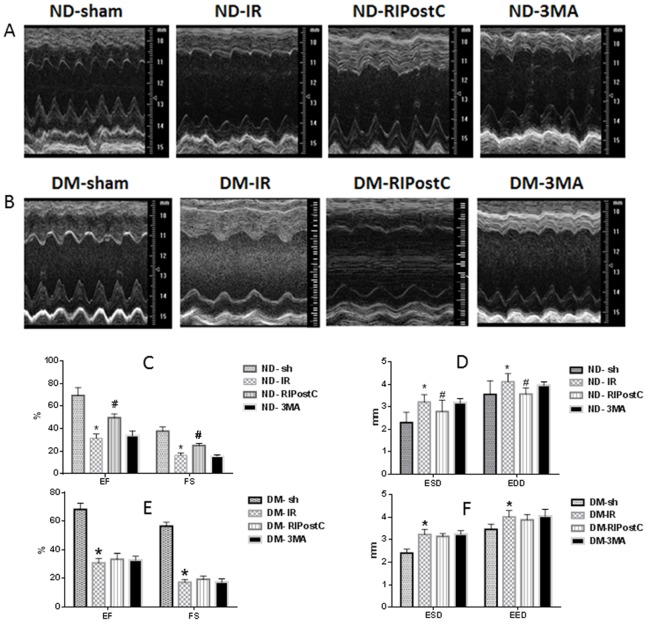
Effect of RIPostC on LV systolic function. (A–B) Representative M-mode echocardiograms recorded from the parasternal short axis on the level of the papillary muscles of the left ventricle (LV) in each group. (C) Ejection fraction (EF) and fractional shortening (FS) were increased significantly in the ND-RIPostC group compared with ND-IR. *P<0.05 vs. ND-IR and ND-sh. ^#^ *P*<0.05 vs. ND-IR and ND-3MA. (D) EED and ESD of LV were significantly reduced in the ND-RIPostC group. *P<0.05 vs. ND-IR and ND-sh. ^#^ *P*<0.05 vs. ND-IR and ND-3MA. (E) EF and FS had no significant difference between DM-RIPostC and DM-IR. (F) EED and ESD of LV were not significantly reduced in the DM-RIPostC group compared with DM-IR. All values are expressed as means ± SEM, n = 5–8 mice per group.

After 3 hrs of IR, echocardiography was performed to determine LV function. RIPostC obviously protected LV systolic function against IR in the ND mice group, but not the DM mice group. As shown in Table S 1 and Figure 2, the ejection fraction (EF) and fractional shortening (FS) was significantly increased in the ND-RIPostC group compared with ND-IR group (49.7±3.46% vs. 31.3±3.95%; 24.96±1.34% vs. 16.18±2.3%, respectively, *P*<0.05 in both cases). Pre-treatment with 3-MA, an autophagy inhibitor, abolished the protective effect of RIPostC. In addition, RIPostC led to an obvious decrease of LV end-diastolic diameter (EDD) and end-systolic diameter (ESD) (EDD 31.3±3.95 mm and ESD 2.79±0.51 mm in ND-RIPostC group, EDD 4.0±0.14 mm and ESD 3.21±0.17 mm in ND-IR group, *P*<0.05 in both cases, Table S 1 and Figure 2). However, in the DM group, there was no significant difference of EF and FS in the DM-RIPostC treated group and the DM-IR group (33.26±4.21% vs. 30.72±3.24%; 19.21±2.35% vs. 17.18±1.95%, respectively, *P*>0.05 in both cases, Table S 2 and Figure 2).

### Effect of RIPostC on Infarct Size and Area at Risk

**Figure 3:**
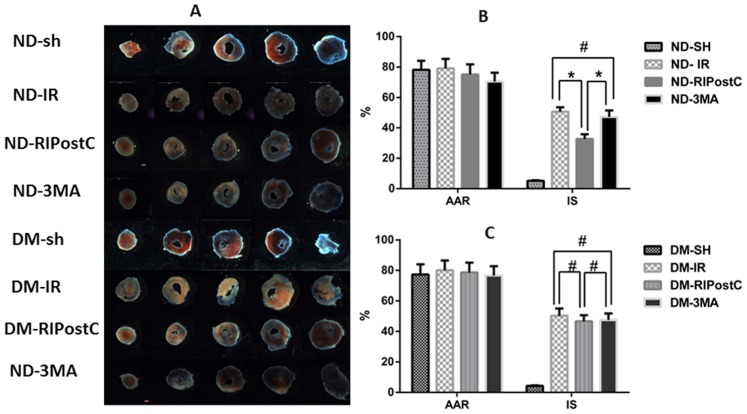
Representative heart cross-sections from each group after IR and staining with TTC to visualize the infarcted area. Representative sections of Evans blue and TTC stained heart following 30-min ischemia and 180-min reperfusion. (B) In ND mice, RIPostC significantly reduced infarct size (IS) compared with ND-IR. However, 3-MA pre-treatment reversed the reduction of IS compared with ND-RIPostC. (C) In DM mice, no significant difference of IS was found between RIPostC group and IR group. The data were expressed as mean ± SEM (n = 8). *P<0.05, ^#^ *P*>0.05.

As shown in Figure 3A and 3B, ND-RIPostC mice has smaller infarct size compared with that in ND-IR mice (32.6±3.0% vs. 50.6±2.4%, *P*<0.05). This cardioprotection effect was reversed by pre-treatment with 3-MA (32.6±3.0% vs. 47.1±4.2%, *P*<0.05). However, compared with mice underwent IR alone in DM animals, RIPostC failed to decrease infarct size in DM animals (46.7±2.7% vs. 50.3±2.1%, *P*>0.05).

### Activation of Autophagy During Myocardial IR

**Figure 4:**
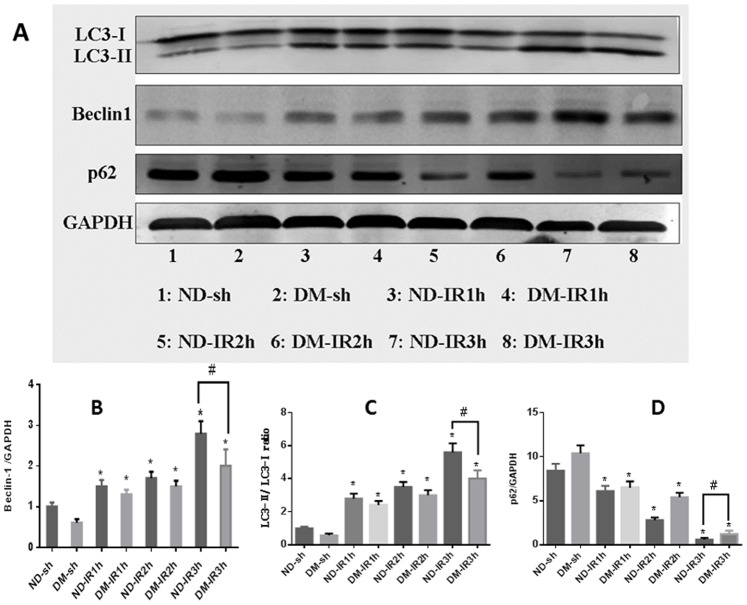
Autophagy was upregulated during myocardial IR. (A) Western blot was performed to test the expression of Beclin-1, LC3, and SQSTM1. (B–D) Bar graph showing the quantification of the immunoreactive band obtained as above. The ratio of LC3-II/LC3-I and the expression of Beclin-1 gradually increased and the SQSTM1 expression gradually decreased. A significant difference of these autophagy markers was detected after 3 hours of postreperfusion in ND group and DM group. Figures are representative images of 5 different heart samples, and each experiment was repeated three times. For ND and DM group, *P<0.05 vs. the Sham group, ^#^ *P*<0.05 (ND-IR3h vs. DM-IR3 h).

**Figure 5:**
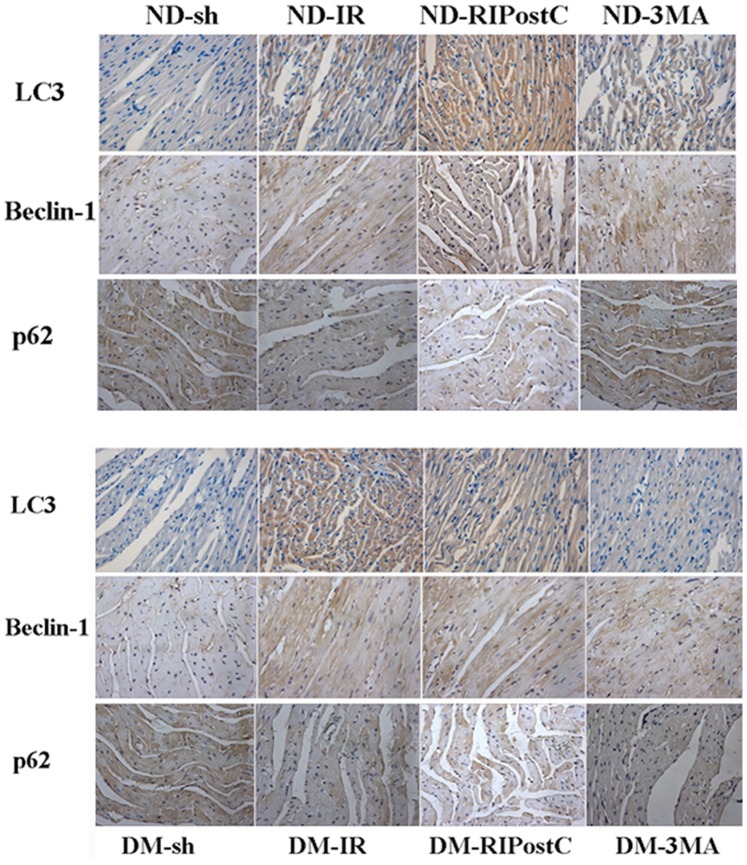
Immunohistochemical analysis of autophagy markers after RIPostC. (A) immunohistochemical analysis was performed after 3 hours of reperfusion to test the expression of autophagic markers. (B–C) Bar graph showed the quantification of the autophagy markers. In the ND groups, RIPostC treatment markedly enhanced the expression of LC3 and Beclin-1, and decreased the level of SQSTM1/p62 compared with IR. (C) No significant difference was observed between DM-RIPostC and DM-IR. Original magnification: ×200. Figures are representative images of at least 4 experiments in each group.

To determine the extent of autophagy, western blot analysis, immunohistochemistry assay, and transmission electron microscopy (TEM) were performed. In western blot analysis, autophagic markers (LC3 and Beclin-1) and SQSTM1/p62 (a substrate of LC3) were detected after 1, 2, and 3 hrs of postreperfusion in both ND and DM groups. Our western blotting results revealed that the ratio of LC3-II/LC3-I and the expression of Beclin-1 gradually increased, and a significant difference after 3 hrs of reperfusion in both ND and DM group (Figure 4 A–C; *P*<0.05 vs. the Sham group). Conversely, the expression of SQSTM1 decreased since the early stage of reperfusion, and a significant reduction was observed after 3 hours of postreperfusion (Figure 4 A and D; *P*<0.05 vs. the Sham group). Autophagy extent in ND group is much greater than that in ND group. A similar autophagy-inducing effect was observed in both ND and DM animals from immunohistochemistry assay (Figure 5).

**Figure 6:**
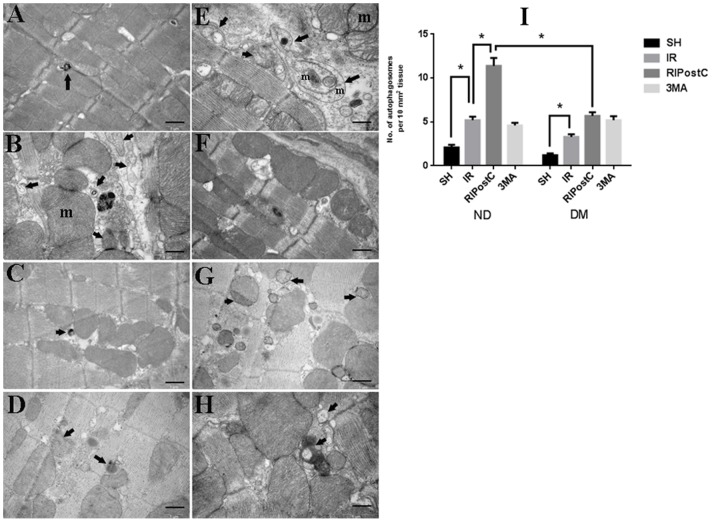
Transmission electron microscopy images to show the ultrastructural changes. A: ND-sh, normal morphology without ultrastructural changes; B: ND-IR, a classic early autophagic vacuole (AV) with double limiting membrane (incompletely closure) containing mitochondria. C: ND-RIPostC, RIPostC-induced autophagosomes (arrows) formation with the characteristic morphology of autophagy and swelling mitochondria. D: ND-3MA, fewer autophagosome. E: DM-sh, normal morphology without ultrastructural changes. F: DM-IR, many autophagosomes contain mitochondria (m). G: DM-RIPostC, few autophagosomes. H: DM-3MA, few autophagosomes with double membrane (arrows) and mitochondrial swelling. Figures are representative images of five different heart samples. Data were presented as means ± SEM, n = 5 per group. *P<0.05 vs. the sh group; ^#^ *P*<0.05 vs. ND-IR and DM-RIPostC. Scale bar = 1 µm.

TEM was also performed to detect autophagic vacuoles (AVs). AVs, including autophagosomes and autophagolysosomes, are generally formed in cells undergoing the autophagic process. Therefore, AVs inside cells is an indicator to analyze the induction extent of autophagy. As shown in TEM images (Figure 6), myocardium sample in the sham operation groups showed normal morphology without ultrastructural changes (Figure 6 A and E), while IR induced oncotic changes in the myocardium with myofibrils disorganization, mitochondrial swelling and cellular lyses (Figure 6B and F). More mature autophagosomes were detected in IR groups compared with that in the sham group in both ND and DM mice (Figure 6).

### Enhancement of Autophagy by RIPostC During Myocardial IR

**Figure 7:**
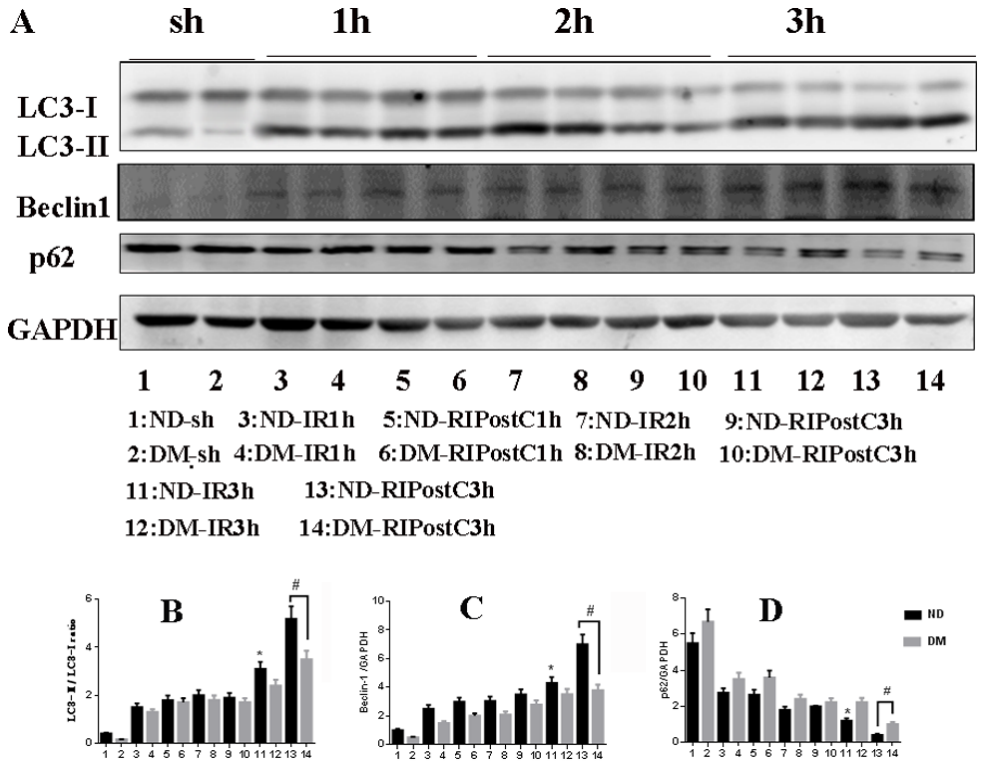
RIPostC induced myocardial autophagy. (A) Western blot was performed after 1, 2, or 3 hours of postreperfusion. (B–D) After 1 or 2 hours of perfusion, RIPostC did not increase the ratio of LC3-II/LC3-I and the expression of Beclin-1 in either ND groups or DM groups. After 3 hours of perfusion, RIPostC increased the ratio of LC3-II/LC3-I and the expression of Beclin-1, decreased SQSTM1 expression in ND group. Data were presented as means ± SEM, n = 5 per group. *P<0.05 vs. ND-RIPostC3 h group, ^#^ *P*<0.05 (ND-RIPostC vs. DM-RIPostC).

As autophagy activity was upregulated significantly after 3 hours of postreperfusion, we evaluated the changes of autophagy under the RIPostC at the same time point by western blot analysis, immunohistochemistry assay and TEM. In ND group, both western blot data (Figure 7) and immunohistochemistry data (Figure 5) showed that, after 3 hours of reperfusion, RIPostC significantly downregulated SQSTM1 level, increased the ratio of LC3-II/LC3-I and the level of Beclin-1 expression compared with animals treated with IR for 3 hours only (Figure 7C; *p<0.05 vs. the IR for 3 hrs group). However, RPIostc failed to exert those autophagy effects in the DM group.

To further clarify the effect of RIPostC on myocardial autophagy during IR, TEM was employed to visualize the autophagosomes in LV myocardium tissue at 3 h time point of IR. Consisting with data from western blot and immunohischemistry assays, we found that in the ND, but not the DM group, RIPostC apparently increased the number of AVs (Figure 6I), suggesting a significant difference of AVs number in the ND-RIPostC group and DM-RIPostC group.

### 3-MA Inhibits Autophagy and Offsets the Cardioprotection Effect of RIPostC in ND Mice

**Figure 8:**
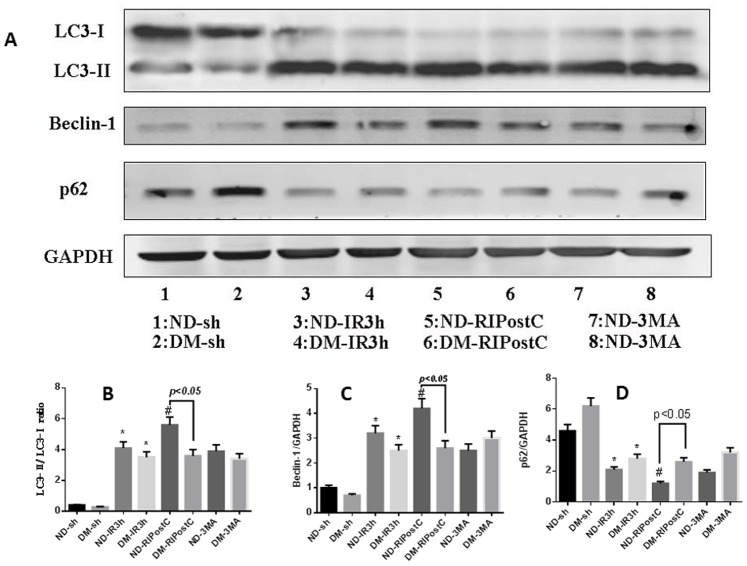
Autophagy inhibitor 3-MA blocked RIPostC autophagy-enhancing effect in ND mice. (A) Western blot was performed to test autophagy extent after 3 hours of postreperfusion in the sham, IR, IR+RIPostC and IR+RIPostC +3MA group. (B–D) Bar graph showed the quantification of the immunoreactive band. In the ND groups, RIPostC upregulated autophagy, 3-MA reversed this upregulation. No similar changes were detected in the DM groups. Data were presented as means ± SEM, n = 5 per group. *P<0.05 vs. the sham group; ^#^ *P*<0.05 vs. the ND-IR group.

To further address the mechanism of RIPostC-induced cardioprotecion, 3-MA, a widely used autophagy inhibitor, was administered before myocardial reperfusion. 3-MA suppresses autophagy by inhibiting Class III phosphatidylinositol 3-kinase (PI3K), whose activity is required for the membrane dynamics involved in autophagic vesicle trafficking [20]. Our echocardiography, I/S, and AAR data showed that 3-MA blocked the protection effect of RIPostC in ND mice (Figure 2 and 3). Consistently, our western blot data (Figure 8; ^#^ *P*<0.05 vs. the ND-IR group), immunohistochemistry data (Figure 5) and TEM data (Figure 6D and 6H) also revealed that 3-MA reversed the pro-autophagy activity of RIPostC in ND mice, but not in DM mice. The ND-RIPostC group has a significantly higher level of autophagy than that in the DM-RIPostC group (Figure 5, Figure 6, and Figure 8).

### Different Effect of RIPostC on Phosphorylation of AMPK in ND and DM Mice

**Figure 9:**
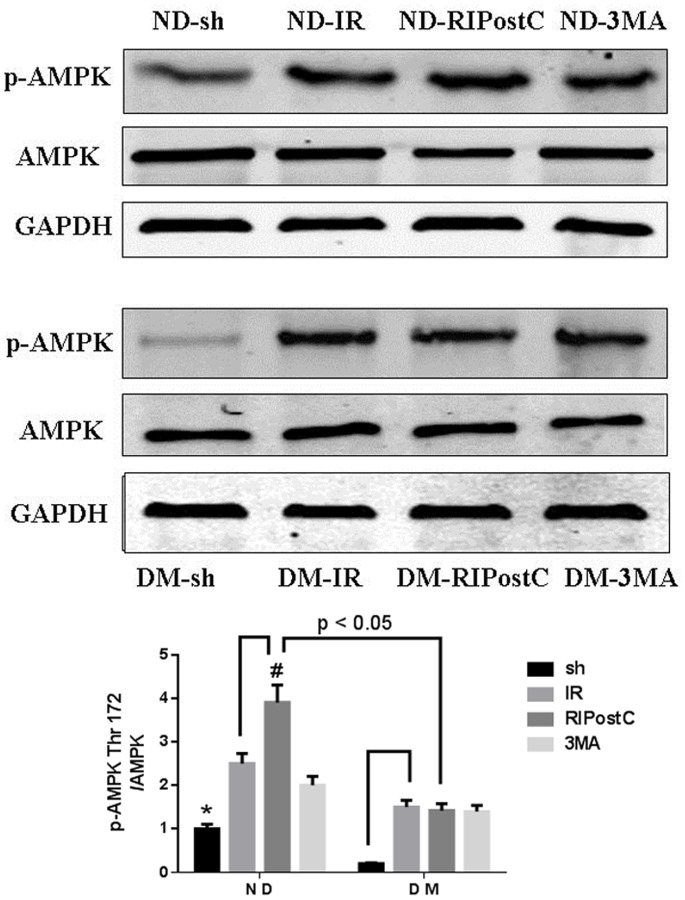
Different effects of RIPostC on phosphorylation of AMPK in ND and DM mice. Western blot was performed to test the level of AMP-activated protein kinase (AMPK) and phospho-AMPK (Thr172). The p-AMPK level in DM-sh group was significantly declined compared with ND-sh group. The p-AMPK level was upregulated in both ND-IR group and DM-IR group. In the ND groups, RIPostC resulted in an increase of p-AMPK expression compared with IR group. In the DM groups, RIPostC did not increase the level of p-AMPK (Figure 9 B). A significant difference of p-AMPK (Thr 172) level was found between ND-RIPostC and DM-RIPotsC. Data were presented as as means ± SEM, n = 5 per group. *P<0.05 vs. ND-IR and DM-sh; ^#^ *P*<0.05 vs. the ND-IR and the DM-RIPostC.

To further elucidate the underlying mechanism of the RIPostC-modulated autophagy, we examined AMPK, a positive regulator of autophagy [22], by detecting its total protein and p-AMPK level after 3 hours of reperfusion. Although no difference of the total AMPK level was detected between the ND-sh group and the DM-sh group, the p-AMPK level in the DM-sh group was significantly lower than that in the ND-sh group (Figure 9). When mice underwent IR, the p-AMPK level was upregulated in both ND-IR group and DM-IR group. In ND, but not DM mice, RIPostC resulted in an increased p-AMPK level compared with the mice treated with IR alone. Taken together, these findings suggested that the cardioprotecion effect of RIPostC might be correlated with the enhancement of AMPK phosphorylation.

## Discussion

This is the first *in vivo* study to explore 1) the autophagy regulation mechanism in RIPostC mediated cardioprotection effect under IR injury, 2) different cardioprotective effect of RIPostC in mice with and without DM under myocardial IR injury. Our study demonstrated that RIPostC, which is delivered by three cycles of 5-min left hinderlimb ischemia followed by 5-min reperfusion, protects cardiac function against IR injury only in ND, but not DM mice. This protective activity is via, at least in part, up-regulating myocardium autophagy. Moreover, AMPK phosphorylation may be involved in this process.

Although autophagy was initially believed to be involved in non-apoptotic form of programmed cell death, the role of autophagy in mediating cell death or survival remains controversial and the underlying signal mechanisms are still obscure. Matsui et al. have shown that, in the case of myocardial ischemia injury, autophagy led to cell survival, whereas the reperfusion injury caused cell death [23]. Recently, Sala-Mercado reported that chloramphenicol succinate induced cardioprotection through up-regulation of autophagy in swine myocardial IR models [12]. Yan et al. discovered that autophagy was more pronounced in the surviving area in chronically ischemic swine myocardium [24]. In current study, we observed RIPostC resulted in the upregulation of LC3-II/LC3-I ratio and Beclin 1 level, and downregulation of SQSTM1/p62 at 3 h time point after reperfusion, demonstrating autophagy activation at the early stage of rerperfusion in normal mice myocardial IR model (Figure 7 and 8). However, in DM mice, RIPostC failed to upregualtion of autophagy. One possible explanation may be that poor autophagy activation or impaired autophagy is a pathological mechanism in myocardial IR model with DM. Because of unpredictable ischaemia-reperfusion syndromes, RIPostC technique has potent translatable clinical application significance than preconditioning strategy. However, to date, little has been known about whether the carioprotective effect of remote postconditioning was related with autophagy induction. Our results provided evidence that the cardioprotection induced by RIPostC may be correlated with up-regulation of autophagy at 3 h time point postreperfusion in ND mice myocardial IR model. In comparison, the STZ-induced DM mice RIPostC failed to further activate autophagy at the same time point. Previously, Qi et al. reported that RIPostC decreased infart size in celebral ischemia injury via inducing autopahgy in the early stage (0 min and 10 min) of reperfusion [25]. However, other conflicting data suggested that postconditioning (not remote postconditoning) alleviated celebral Ischemia injury through inhibiting autophagy. The discrepancy of these studies may depend on the type of postconditioning, extent of autophagy, timing (ischemia vs. reperfusion), and the threshold for ischemia conditioning stimuli [26]. In the STZ-induced DM mice model, we found that RIPostC did not result in a decrease in myocardial infarct size. Interestingly, our current study supports Badalzadeh’s finding, which demonstrated that ischemic postconditoning failed to protect the STZ-induced diabetic myocardium against IR injury [15]. Kristiansen also reported ischaemic preconditioning does not protect heart from type 2 diabetic animals [27]. Consistent with other studies [26], we also found no cardioprotection evoked by RIPostC and attenuated autophagy in DM animals.

To further clarify the contribution of the autophagy mechanism to RIPostC-induced cardioprotection in ND mice, 3-MA, a widely used inhibitor of autophagy, was applied before postconditioning. Within the DM groups, 3-MA administration before reperfusion abolished RIPostC-induced cardioprotection and downregulated the autophagic activity, implicating that autophagic pathway might play a vital role in RIPostC-induced cardioprotection in DM IR mice, which is consistent with other study [11].

To further elucidate the mechnism of RIPostC effect, we examined autophagy induction by RIPostC during myocardial IR in ND mice and DM mice. No difference of autophagy activity was observed between the ND-RIPostC group and DM-RIPostC group at 1 and 2 h time point after reperfusion. However, at 3 h time point, the ratio of LC3-II/LC3-I and the protein level of Beclin-1 and SQSTM1/p62 showed significant difference between two groups, suggesting RIPostC stimuli have time dependent effect on autophagy activation (Figure 7). The mechanism of this time dependent autophagy activation need to be resolved in future investigation.

The present study provides a novel finding which enhances our understanding on the discrepancy of RIPostC mediated cardioprotection effect on myocardial IR injury with and without diabetes. Adenosine 5′-monophosphate-activated protein kinase (AMPK) is a sensor of energy molecule ATP, and is activated when the ratio of ATP/ADP is decreased during exercise, hypoxia, oxidative stress and glucose deprivation. Intrinsic AMPK activation plays a vital role in the stress response to myocardial ischemia and hypertrophy [28]. In addition, AMPK is a positive regulator of autophagy. The protein level of phosphorylated AMPKα (Thr-172) was reduced in the diabetic heart, suggesting an inhibited AMPK signaling pathway as reported before [22]. However, the link among RIPostC, intrinsic AMPK and cardiac autophagy is not determined. Recently, Bouhidel reported that, although ischemia postconditioning reduced myocardial infarct size in healthy animals, it failed to induce cardioprotection in ob/ob mice compared with wild mice. The lack of enhanced phosphorylation by ischemia postconditioning of Akt, ERK1/2, and AMPK may partially explain the loss of cardioprotection in diabetic mice model [29]. Consistent with these results, our data indicated that, in the baseline level of DM mice, the activity of pAMPK is down-regulated as compared with non-diabetic mice. Our data were also supported by Xu’s study, in which diabetes activates Akt and mTOR while inhibites AMPK signaling in the heart [30]. In addition, our data demonstrated that myocardial IR significantly upregulated p-AMPK (Thr172) level in both ND groups and DM groups, suggesting that the activation of AMPK plays a vital role in myocardial IR injury. Interestingly, compared with the DM group, RIPostC further activated AMPK in ND group (Figure 9), suggesting that RIPostC-induced AMPK activation may be involved in the cardioprotection effect.

However, Venna recently reported that ischemia preconditioning (IPC) delivered by a brief 3-min middle celebral artery occlusion induces endogenous neuroprotection from a subsequent ischemic injury, and the beneficial effect occurred in parallel with a significant inhibition of pAMPK protein expression, which suggesting that AMPK plays an important role in IPC-mediated neuroprotection [31]. The discrepancy of AMPK expression and its interaction with protective effect of ischemia conditioning might be explained by the threshold and time window of ischemia conditioning stimuli, species specific, and organ specific [26]. Some compelling research has provided evidence that AMPK is a positive regulator of autophagy [22], [32]. The discrepancy of AMPK phosphorylation may affect the downstream signaling such as eNOS-mTOR pathway [33], which may result in a discrepancy of autophagy activation between ND and DM animals. Furthermore, many other pieces of evidence has suggested that the development of insulin resistance, which is the hallmark in diabtetic mellitus or metabolic-syndrome, is associated with progressive changes of myocardial autophagy, apoptosis and inflammation [34]. Interestingly, Xie reported that the improvement of cardiac functions by chronic metformin treatment is correlated with the enhanced cardiac autophagy in diabetic OVE26 mice [35]. Further studies are needed to clarify the molecular mechanism contributing to the loss of cardioprotective effect of RIPostC in myocardial IR model with DM.

In conclusion, our study suggested that myocardial protection invoked by RIPostC in normal mice might be mediated in part via up-regulation of autophagy. The loss of cardioprotive effect induced by RIPostC on myocardial IR in diabetes mice might be association with the under-induction of myocardium autophagy.

## Supporting Information

Table S1 **Echocardiography parameters of the ND group.**
(DOC)

Table S2 **Echocardiography parameters of the DM group.**
(DOC)
