# Supplementary material for: Autophagy Is Involved in the Cardioprotection Effect of Remote Limb Ischemic Postconditioning on Myocardial Ischemia/Reperfusion Injury in Normal Mice, but Not Diabetic Mice
Source: PLoS One. 2014 Jan 23;9(1):e86838. doi: 10.1371/journal.pone.0086838 (PMC3900658; doi:10.1371/journal.pone.0086838)
Supplement: Table S1 — Echocardiography parameters of the ND group. (DOC) [file pone.0086838.s001.doc]

**Supporting Information Legends**

**Table S 1** Echocardiography parameters of the ND group

|  | ND-sh (n=5) | ND-IR(n=8) | ND-RIPostC(n=8) | ND-3MA(n=8) |
| --- | --- | --- | --- | --- |
| Heart rate (beats/min) | 496±14 | 457±23* | 454±20 | 450±19 |
| End-systolic diameter (mm) | 2.3±0.46 | 3.21±0.17* | 2.79±0.51‡ | 3.18±0.19#$ |
| End-diastolic diameter (mm) | 3.56±0.6 | 4.0±0.14* | 3.57±0.28‡ | 3.96±0.16#$ |
| FS (%) | 37.75±4.82 | 16.18±2.30* | 24.96±1.34‡ | 15.52±1.15#$ |
| EF (%) | 69.57±6.85 | 31.30±3.95* | 49.70±3.46‡ | 33.8±4.17#$ |

Results are presented as mean ± SEM. *p<0.05 vs ND-sham, ‡p<0.05 vs ND-IR,

# p<0.05 vs ND-RIPostC, $p>0.05 vs ND-IR.
