# Supplementary material for: Autophagy Is Involved in the Cardioprotection Effect of Remote Limb Ischemic Postconditioning on Myocardial Ischemia/Reperfusion Injury in Normal Mice, but Not Diabetic Mice
Source: PLoS One. 2014 Jan 23;9(1):e86838. doi: 10.1371/journal.pone.0086838 (PMC3900658; doi:10.1371/journal.pone.0086838)
Supplement: Table S2 — Echocardiography parameters of the DM group. (DOC) [file pone.0086838.s002.doc]

**Table S** 2 Echocardiography parameters of the DM group

|  | DM-sh(n=5) | DM –IR(n=8) | DM RIPostC(n=8) | DM -3MA(n=8) |
| --- | --- | --- | --- | --- |
| Heart rate (beats/min) | 502±11 | 464±16* | 471±19 | 453±13 |
| End-systolic diameter (mm) | 2.41±0.18 | 3.24±0.11* | 3.15±0.12‡ | 3.25±0.09#$ |
| End-diastolic diameter (mm) | 3.47±0.23 | 4.02±0.14* | 3.89±0.13‡ | 4.05±0.08#$ |
| FS (%) | 36.53±2.78 | 17.1±1.95* | 19.21±2.35‡ | 17.24±2.41#$ |
| EF (%) | 68.23±4.32 | 30.72±3.24* | 33.26±4.21‡ | 32.68±4.73#$ |

Results are presented as average mean ± SEM. *p<0.05 vs DM-sham, ‡p>0.05 vs DM-IR,

#p>0.05 vs DM-RIPostC, $p>0.05 vs DM-IR.
